# The expression of genes coding for distinct types of glycine-rich proteins varies according to the biology of three metastriate ticks, *Rhipicephalus *(Boophilus) *microplus*, *Rhipicephalus sanguineus *and *Amblyomma cajennense*

**DOI:** 10.1186/1471-2164-11-363

**Published:** 2010-06-08

**Authors:** Sandra R Maruyama, Elen Anatriello, Jennifer M Anderson, José M Ribeiro, Lucinda G Brandão, Jesus G Valenzuela, Beatriz R Ferreira, Gustavo R Garcia, Matias PJ Szabó, Sonal Patel, Richard Bishop, Isabel KF de Miranda-Santos

**Affiliations:** 1 Departament of Biochemistry and Immunology, Ribeirão Preto School of Medicine, University of São Paulo, Ribeirão Preto, SP 14049-900, Brazil; 2 Laboratory of Malaria and Vector Research, National Institute of Allergy and Infectious Diseases, National Institutes of Health, Bethesda, MD 20892-8132, USA; 3 Departament of Maternal and Child and Public Health Nursing, Ribeirão Preto School of Nursing, University of São Paulo, Ribeirão Preto, SP 14049-900, Brazil; 4 School of Veterinary Medicine, Federal University of Uberlândia, Uberlândia MG 38400-902, Brazil; 5 International Livestock Research Institute, Nairobi, Kenya; 6 Embrapa Recursos Genéticos e Biotecnologia, 70770-900, Brasília, DF, Brazil; 7 Universidade Paulista, Avenida Baguaçu, 1939, 16018-280 - Araçatuba, SP - Brasil

## Abstract

**Background:**

Ticks secrete a cement cone composed of many salivary proteins, some of which are rich in the amino acid glycine in order to attach to their hosts' skin. Glycine-rich proteins (GRPs) are a large family of heterogeneous proteins that have different functions and features; noteworthy are their adhesive and tensile characteristics. These properties may be essential for successful attachment of the metastriate ticks to the host and the prolonged feeding necessary for engorgement. In this work, we analyzed Expressed Sequence Tags (ESTs) similar to GRPs from cDNA libraries constructed from salivary glands of adult female ticks representing three hard, metastriate species in order to verify if their expression correlated with biological differences such as the numbers of hosts ticks feed on during their parasitic life cycle, whether one (monoxenous parasite) or two or more (heteroxenous parasite), and the anatomy of their mouthparts, whether short (Brevirostrata) or long (Longirostrata). These ticks were the monoxenous Brevirostrata tick, *Rhipicephalus *(Boophilus) *microplus*, a heteroxenous Brevirostrata tick, *Rhipicephalus sanguineus*, and a heteroxenous Longirostrata tick, *Amblyomma cajennense*. To further investigate this relationship, we conducted phylogenetic analyses using sequences of GRPs from these ticks as well as from other species of Brevirostrata and Longirostrata ticks.

**Results:**

cDNA libraries from salivary glands of the monoxenous tick, *R. microplus*, contained more contigs of glycine-rich proteins than the two representatives of heteroxenous ticks, *R. sanguineus *and *A. cajennense *(33 versus, respectively, 16 and 11). Transcripts of ESTs encoding GRPs were significantly more numerous in the salivary glands of the two Brevirostrata species when compared to the number of transcripts in the Longirostrata tick. The salivary gland libraries from Brevirostrata ticks contained numerous contigs significantly similar to silks of true spiders (17 and 8 in, respectively, *R. microplus *and *R. sanguineus*), whereas the Longirostrata tick contained only 4 contigs. The phylogenetic analyses of GRPs from various species of ticks showed that distinct clades encoding proteins with different biochemical properties are represented among species according to their biology.

**Conclusions:**

We found that different species of ticks rely on different types and amounts of GRPs in order to attach and feed on their hosts. Metastriate ticks with short mouthparts express more transcripts of GRPs than a tick with long mouthparts and the tick that feeds on a single host during its life cycle contain a greater variety of these proteins than ticks that feed on several hosts.

## Background

In order to acquire a blood meal, Ixodid (hard) ticks secrete diverse salivary proteins that inhibit their hosts' defense mechanisms and permit hematophagy to proceed for many days [1]. But ticks must first attach to the skin of their hosts and attachment must be effective for the duration of the tick's blood meal. Ixodid ticks are classified by the number of different hosts they feed on during the parasitic phase of their life cycle; one host, two hosts or three hosts. Ticks that complete the entire parasitic cycle on one host are monoxenous parasites, whereas tick that feed on two or more different hosts with an interval off the host between the feeds are heteroxenous. Success of attachment on one or more hosts depends, among other factors, on the salivary proteins that are believed to form cement cones. These structures fix tick mouthparts to the host's skin and possibly disguise and/or lubricate them [2]. The architecture of the cement cone depends on the both the depth of penetration of the tick's hypostome into the host's skin and the degree to which cement encases the hypostome. The cattle tick, *Rhipicepahalus microplus*, and the brown dog tick, *R. sanguineus*, are classified as Brevirostrata ticks because their mouthparts are short and barely penetrate into the epidermis of theirs hosts. These parts are therefore assisted by a wide cement cone that reaches more deeply into this layer of skin and also extrudes the epidermis [3]. Consequently, the cement cone of Brevirostrata ticks tends to be wide and deep, completely surrounding the hypostome and extruding above the epidermis of the host skin [4]. Histological cross-sections of an adult *R. sanguineus *attached to a dog clearly illustrate the superficial penetration of the hyposotome and the extensive cement cone which appears to "glue" the mouthparts in place [5]. *R. microplus *is a monoxenous tick and its life cycle, spent on a single host, is of approximately 21 days; *R. sanguineus *is a heteroxenous tick. Conversely, *Amblyomma cajennense*, also a heteroxenous parasite, is a Longirostrata tick with its long hypostome fully penetrating well into its host's dermis and encased by a narrow cement cone [3]. Several salivary proteins present in the tick's cement cone are rich in glycine (glycine-rich proteins, GRPs) [3,6,7]. GRPs are abundant in nature and constitute a large family of heterogeneous proteins enriched in glycine residues by various proportions, occupying from 20% to 70% of the total amino acid residues of the protein. GRPs can be classified into several groups based on their molecular structure [8,9].

During the course of our studies of the transcriptome of salivary glands from *R. microplus*, *R. sanguineus *and *A. cajennense *we annotated different types of GRPs and observed that these contigs represent from 3 to over 6% of the total number, higher than any other class of protein. Furthermore, we observed that the distribution and abundance of the contigs and the number of transcripts that form them differed according to the species. Since proteins isolated from the cement cone are rich in glycine and this structure may have a role in attachment and since the various species of ticks have different requirements for attachment, herein we perform initial tests of the hypothesis that there are not only anatomical, but also chemical differences between the cement cones produced by these three species of ticks. These differences could vary according to their biology, such as whether they infest one or more hosts and whether anatomy of their mouthparts comprises short or long hypostomes.

We constructed three non-normalized, PCR-based cDNA libraries from the salivary glands of female *R. sanguineus*, *R. microplus *and *A. cajennense *and analyzed the expressed sequence tags (ESTs) obtained using customized bioinformatics software. We observed that the expression of contigs and their transcripts coding for glycine-rich proteins differed in quantity as well in diversity, depending on the species of the tick. In order to further test this hypothesis we also performed a phylogenetic analysis using the sequences from our work as well as of publicly available sequences from all the available sialomes of other species of heteroxenous and Longirostrata or Brevistrata ticks that have been annotated as GRPs.

## Results and Discussion

### Library Construction

A total of 1440 plaque phages were sequenced from each of the three salivary gland libraries to generate 5' Expressed Sequence Tags (ESTs). A total of approximately 2,900 high quality sequences, including 1,152 from the salivary glands of female *R. microplus *(SGFRm), 824 from salivary glands of female *R. sanguineus *(SGFRs) and 929 from salivary glands of female *A. cajennense *(SGFAc). Redundant sequences were clustered into related groups using BLASTN and then assembled into contiguous sequences yielding 1,406 unique contigs of which 245 were derived from two or more ESTs (transcripts) and 1,165 were derived from a single EST (singleton). As seen in Table 1, GRPs are abundantly expressed in the salivary glands, ranging from 3- 6% of the total contigs sequenced from these libraries. The SGFRm library contained more ESTs similar to genes coding for GRPs than the other two libraries. The SGFRm and SGFRs libraries exhibit a similar number of ESTs for GRPs (57 and 47 ESTs, respectively), but comparing the number of unique contigs similar to GRPs, SGFRm contained almost double the number (n = 33) of unique contigs as SGFRs (n = 16) and triple that of SGFAc (n = 11). This finding shows that saliva of *R. microplus*, a Brevirostrata, one-host tick, contains twice as many different GRPs than the other two species of ticks examined herein, one a Brevirostrata, three host tick, the other a Longirostrata, three host tick. The SGFAc library contained approximately the same proportion of unique GRP contigs as the SGFRs library, however these are formed by fewer ESTs (23) relative to the other two libraries (57 and 47 ESTs, from SGFRm and SGFRs, respectively).

**Table 1:** Characteristics cDNA libraries constructed using salivary glands (SG) dissected from three feeding female Ixodid ticks: *Rhipicephalus sanguineus *(Rs), *Rhipicephalus (Boophilus) microplus *(Rm) *a*nd *Amblyomma cajennense *(Ac).

Library	ESTs	Contigs	ESTs/Transcripts	ESTs encoding GRPs	Contigs of GRPs	Average n° GRP ESTs/Contig of GRP
SGFRm	1,152	533	2.16	57 (4.94)^a^	33 (6.19)^a^	1.72
SGFRs	874	455	1.92	47 (5.37)^a^	16 (3.51)^a^	2.93
SGFAc	929	418	2.22	23 (2.90)^a^	11 (3.82)^a^	2.09

^a ^Percent of total

### Comparison of library-derived glycine rich proteins to published and custom databases

Comparsion of the contigs from the three libraries with a customized database of all Arachnida proteins found in Genbank revealed that 60 contigs had similarities with 21 different types of GRP, based on published annotated sequences (Table 2). Contigs were considered to encode GRPs if the translated amino acid sequence contained a glycine content of at least 20% (with three exceptions among the 60 unique contigs, which contained 11 and 17% glycine). The most abundant GRP (41 total ESTs) found among all three libraries was a 506 amino acid protein containing 25% glycine obtained from *R. haemaphysaloides *and annotated as "unknown function". Flagelliform silk proteins (~50% glycine), identified from various spider species, was the second most abundant GRP found among the three libraries (n = 23 ESTs). Proteins annotated as cement and cement-like proteins from *H. longicornis, I. scapularis *and *R. appendiculatus *were also commonly observed among the three libraries (Table 2).

**Table 2:** Description of matches with glycine-rich proteins present in the Arachnida protein database for transcripts from *A. cajennense, R. sanguineus *and *R. microplus*

Best Match to Arachnida Database	Accession number of Best Match	Size (amino acid)	% Glycine of Best match	Library	Transcript name (Number of ESTs)	% Glycine in respective transcript*	E-value of Match
cement-like antigen [*H. longicornis*]	gi 116642505	179	34,63	SGFAc	Ac147 (1)	28,81	9E-005
				SGFRs	Rs345 (1)	21,78	5E-009
				SGFRm	Rm62 (1)	21,11	5E-009
				SGFRm	Rm115 (2)	22,91	1E-011
				SGFRm	Rm 519 (1)	23,37	3E-007
				SGFRm	Rm61 (3)	32,2	1E-020
cement-like antigen [*H. longicornis*]	gi 125597020	217	38,7	SGFRm	Rm265 (1)	22,22	1E-009
				SGFRm	Rm71 (1)	41,32	1E-017
				SGFRm	Rm470 (1)	31,79	
NPL-2 [*I. pacificus*]	gi 51011404	78	38,46	SGFRm	Rm234 (1)	21,35	1E-006
putative cement protein [*I. scapularis*]	gi 50363174	119	50,42	SGFRm	Rm388 (1)	20,43	5E-007
putative cement protein RIM36 [*R. appendiculatus*]	gi 21885262	334	24,55	SGFAc	Ac52 (2)	20,57	8E-024
				SGFRm	Rm77 (1)	25,82	2E-009
salivary gland-associated protein 64P [*R. appendiculatus*]	gi 20069012	154	29,22	SGFAc	Ac109 (1)	20	4E-012
				SGFRs	Rs12 (13)	30,43	1E-025
Unknown [*R.haemaphysaloides*]	gi 45479213	506	25,29	SGFAc	Ac9 (10)	24,8	2E-028
				SGFRs	Rs17 (5)	22,05	1E-149
				SGFRs	Rs19 (1)	20,31	7E-062
				SGFRs	Rs20 (1)	21,1	3E-082
				SGFRs	Rs156 (1)	30,37	1E-020
				SGFRs	Rs402 (1)	25,77	2E-028
				SGFRs	Rs428 (1)	30,72	2E-028
				SGFRm	Rm29 (11)	36,94	3E-060
				SGFRm	Rm70 (2)	31,19	2E-024
				SGFRm	Rm30 (1)	30,51	2E-020
				SGFRm	Rm31 (1)	26,08	3E-022
				SGFRm	Rm67 (3)	20,38	3E-074
				SGFRm	Rm85 (2)	11,34	3E-025
				SGFRm	Rm479 (1)	23,76	4E-010
acanthoscurrin 1 precursor [*A. gomesiana*]	gi 27524417	156	62,17	SGFAc	Ac354 (1)	42,85	3E-014
				SGFRs	Rs26 (6)	31,95	2E-019
flagelliform silk protein [*A. trifasciata*]	gi 13561982	1.002	56,78	SGFAc	Ac233 (1)	30,47	3E-005
				SGFRm	Rm259 (1)	22,22	1E-005
flagelliform silk protein [*A. trifasciata*]	gi 13561980	651	47,31	SGFRm	Rm533 (1)	30,91	1E-017
flagelliform silk protein [*N.clavipes*]	gi 2833647	871	46,84	SGFRs	Rs54 (3)	33,14	9E-020
				SGFRm	Rm35 (1)	20,68	2E-011
				SGFRm	Rm36 (1)	22,53	8E-010
				SGFRm	Rm32 (3)	25	4E-018
flagelliform silk protein [*N.clavipes*]	gi 7106224	2.249	54,37	SGFRs	Rs130 (1)	21,42	4E-004
				SGFRm	Rm34 (3)	25,54	7E-012
				SGFRm	Rm129 (2)	26,82	5E-012
flagelliform silk protein [*N. madagascariensis*]	gi 7106228	1.884	51,8	SGFRm	Rm33 (2)	27,48	3E-021
				SGFRm	Rm37 (1)	21,64	7E-011
flagelliform silk protein [*N. madagascariensis*]	gi 7106229	626	47,12	SGFRm	Rm76 (2)	17,22	7E-008
				SGFRm	Rm197 (1)	25	2E-004
fibroin 2 [*D. spinosa*]	gi 89113990	623	35,63	SGFRs	Rs29 (5)	23,92	6E-011
				SGFRm	Rm433 (1)	23,8	1E-005
major ampullate spidroin 1 [*L. hesperus*]	gi 89276817	1.065	41,22	SGFRm	Rm324 (1)	48,38	1E-019
major ampullate dragline silk protein-2 [*Araneus ventricosus*]	gi 27228959	429	27,03	SGFAc	Ac13 (3)	32,46	3E-009
major ampullate spidroin 2-1 [*K. hibernalis*]	gi 47007938	185	47,02	SGFAc	Ac15 (1)	35,92	2E-013
				SGFRm	Rm381 (1)	22,97	7E-017
				SGFRm	Rm418 (1)	21	9E-008
major ampullate spidroin 2-2 [*K. hibernalis*]	gi 47007952	214	49,53	SGFAc	Ac14 (1)	32,33	3E-025
					Ac17 (1)	32,09	2E-010
					Ac16 (1)	31,96	1E-014
				SGFRs	Rs38 (1)	17,35	1E-010
				SGFRs	Rs37 (3)	31,09	6E-018
				SGFRs	Rs70 (2)	31,65	1E-022
spidroin 1 [*N. clavipes*]	gi 2911274	544	40,25	SGFRs	Rs84 (2)	45,23	2E-004
SPD1_NEPCL Spidroin-1 [*Dragline silk fibroin 1*]	gi 1174414	747	42,3	SGFRm	Rm185 (1)	26,25	4E-005

* Computation of glycine content was calculated based on translated consensus sequence

Comparing the BLAST results of the three libraries shows that, with 33 contigs representing 57 ESTs, *R. microplus *contained the most abundant contigs homologous to GRPs as compared to 11 contigs from *A. cajennenes *and 16 contigs from *R. sanguineus*. Salivary glands from *R. microplus *also contained the greatest variety of GRP with contigs homologous to 11 different GRPs whereas *A. cajennense *and *R. sanguineus *salivary glands contained 9 and 8 different GRPs, respectively (Table 2).

### Differential expression of GRPs in Brevirostrata and Longistrata, and monoxenous and heteroxenous ticks

**Figure 1:**
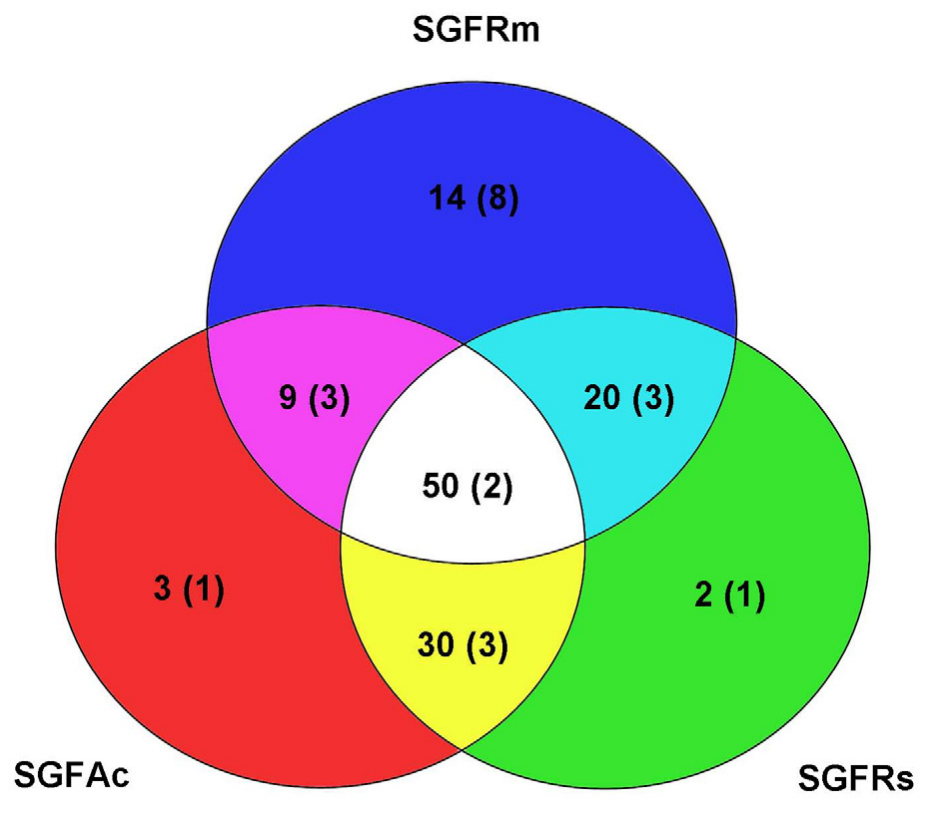
**Venn diagram showing the distribution of glycine rich protein (GRP) ESTs among three species of hard ticks, *A. cajennense, R. sanguineus *and *R. microplus***. Numbers in parentheses represent types of GRP according to the classification in Table 2.

As reported above, the distribution of GRPs in three Ixodid ticks differed according to the species. In order to better display this distribution, Figure 1 shows a Venn diagram of the numbers of GRP ESTs and types of GRPs (numbers in parentheses) found in common among the three species of ticks studied herein. Figure 1 also shows the number of GRP ESTs and types of GRPs that are unique to each species. More GRP ESTs are expressed uniquely in the salivary glands of females of *R. microplus *(14 versus 3 and 2 ESTs for, respectively *A. cajannense *and *R. sanguineus*). These transcripts represented 8 unique types of GRPs in *R. microplus*, whereas *R. sanguineus *and *A. cajannense *each presented only unique 1 type of GRP. On the other hand, only two GRPs (from a total of 21 types) were common to all three species of ticks and were represented by 50 ESTs (Figure 1 and Table 2). We also analyzed the distribution of 21 types of GRPs among the three libraries. As shown in Figure 1, *R. microplus *contained almost twice as many types of GRPs than *R. sanguineus *or A. *cajennense *[SGFRm: 16 types of GRPs; SGFRs and SGFAc: 9 types of GRPs each (see numbers in parentheses)]; *R. microplus *contains twice the amount of contigs encoding GRPs (SGFRm, SGFRs and SGFAc contained 33, 16 and 11 contigs of GRPs, respectively).

A protein previously described in *Rhipicephalus haemaphysaloides haemaphysaloides *(gi 45479213), annotated as of "unknown function", represented the class of GRP with which the majority of ESTs in the 3 libraries presented similarity (Table 2). Over half of these transcripts derived from the library from *R. microplus *salivary glands (21 from SGFRm, 10 from SGFRs and 10 from SGFAc). Interestingly, the SGFRm library does not present any EST with similarity to salivary gland-associated protein 64P from *Rhipicephalus appendiculatus *ticks (gi 20069012), at least on the first 10 best hits, contrary to what was found for SGFRs and SGFAc. 64P is a GRP of interest because it is a potentially protective antigen for some species of host [7,10]. The amino acid sequence of the 64P secreted salivary protein is similar to epidermal/dermal keratin and collagen proteins, which are mammalian structural proteins of the skin [8,11], and salivary homologues are present in several species of ticks [7].

As noted above, the annotation of the three cDNA libraries using the BLAST results permitted us to observe that the libraries and some contigs contained fewer or more transcripts and sequences coding for proteins similar to GRPs than expected from a random distribution, as evaluated with the χ^2 ^or Fisher exact tests. Table 3 presents the distribution of all transcripts coding for the GRPs observed among the three salivary gland libraries from females of the tick species studied herein, *R. microplus*, *R. sanguineus *and *A. cajennense*. SGFRm and SGFRs libraries contain significantly (*P *= 0.006 and *P *= 0.003, respectively; χ^2 ^test) more transcripts coding for all types of GRPs than the SGFAc library; transcripts for GRPs were equally represented in the SGFRm and SGFRs libraries (P = 0.821, χ^2 ^test). These results show that for Brevirostrata ticks, *R. microplus *and *R. sanguineus*, the expression in salivary glands of all types of GRPs was significantly higher than in those of a Longirostrata tick (*A. cajennense*). The *R. microplus *salivary gland library contained more types and transcripts of proteins similar to GRPs than the SGFRs, but the difference in the proportions of the GRPs did not quite reach significance with the present depth of sequencing (P = 0.072, χ^2 ^test; Table 3). Nevertheless, these results show that a Brevirostrata, monoxenous tick, which remains attached to the same host for at least three weeks, relies on a greater variety of GRPs than the Brevirostrata heteroxenous tick examined in this study. On the other hand, the library from *R. microplus *contained significantly (*P *= 0.015, χ^2 ^test) more transcripts of GRPs than the library from the salivary glands of the Longirostrata, heteroxenous tick, *A. cajennense *(Table 3). This finding suggests that in order to feed on a single host for up to three weeks, monoxenous ticks with short mouthparts must be equipped to deal with a larger repertoire of the host's local homeostatic mechanisms. It is noteworthy that by the time the monoxenous tick *R. microplus *completes its blood meal its host will have mounted an adaptive immune response. The greater diversity of GRPs in this species may reflect a form of antigenic variation.

**Table 3:** Differential Abundance of Transcripts and Diversity of Types of GRPs in Salivary Gland Libraries from females of *Rhipicephalus *(Boophilus) *microplus *SGFRm), *Rhipicephalus sanguineus *(SGFRs)and *Amblyomma cajennense *(SGFAc)

Representation of Transcripts Coding for GRPs							
**Library**		**N° of ESTs**		**Library**	**N° of ESTs**		*P *value*
				
		**Observed**	**Expected**		**Observed**	**Expected**	
SGFRm		57	59.620	SGFRs	47	45.380	P = 0.821
SGFRm		57	45.333	SGFAc	23	35.667	*P = 0.006*
SGFRs		47	34.421	SGFAc	23	35.579	*P = 0.003*

**Representation of Transcripts Coding for GRPs**							

**Library**		**N° clusters**		**Library**	**N° clusters**		*P *value*
					
		**Observed**	**Expected**		**Observed**	**Expected**	
SGFRm		33	27.312	SGFRs	16	22.688	P = 0.072
SGFRm		33	25.617	SGFAc	11	19.383	*P = 0.015*
SGFRs		16	14.130	SGFAc	11	12.870	P = 0.592

**Representation of the Most Abundant Transcripts Coding for Specific Types of GRPs**							

**Best match to NR protein database**	**Library**	**N° of ESTs**		**Library**	**N° of ESTs**		***P *value**
				
		**Observed**	**Expected**		**Observed**	**Expected**	
gi|45479213|unknown *Rhipicephalus haemaphysaloides*	SGFRm	21	17.678	SGFRs	10	13.322	P = 0.372
	SGFRm	21	17.217	SGFAc	10	13.783	P = 0.232
Acanthoscurrin	SGFRs	6	3,301	SGFAc	1	3,699	P = 0.424
64P	SGFRs	13	9.432	SGFAc	1	4.568	*P = 0.002*
Silk-like proteins from true spiders	SGFRm	23	22,749	SGFRs	17	17,251	P = 0.936
	SGFRm	23	17,247	SGFAc	8	13,753	P = 0.056
	SGFRs	17	12,185	SGFAc	8	12,815	P = 0.396

*Chi-square test for representation of the glycine-rich proteins distributed in the three cDNA libraries.

We also observed that the distribution of ESTs within some contigs was greater in a given species of tick. Contig 29 from SGFRm, coding for a protein similar to an "unknown" protein from *R. h. haemaphysaloides *ticks (Genbank accession: gi 45479213), was the most abundant transcript among the three libraries and the most abundant in the SGFRm library, with 21 ESTs versus 10 ESTs in both the SGFRs and SGFAc libraries, however it was not differentially represented among the three ticks (Table 3). A contig coding for a GRP similar to "acanthoscurrin 1 precursor" from *Acanthoscurria gomesiana *spiders (Genbank accession gi 27524417) was also not significantly differentially represented (Table 3), although SGFRs contains 6 ESTs and SGFAc has just 1 EST. However, the GRP similar to "salivary gland-associated protein 64P" from *R. appendiculatus *(a Brevirostrata, heteroxenous tick), regarded as a cement protein, was significantly more expressed in salivary glands of female *R. sanguineus *than in those of *A. cajennense *(*P *= 0.001, χ^2 ^test). This result suggests that females of a Brevirostrata, heteroxenous tick rely more on this protein to attach and feed on their last host than Longirostrata, heteroxenous ticks. Finally, regarding the nature of similarities, it was interesting to note that *R. microplus*, *R. sanguineus *and *A. cajennense *expressed, respectively, 23, 17 and 8 transcripts that were similar to silks of true spiders (Araneomorphae; Table 2), however the differences in distribution did not reach statistical significance.

Our results do not preclude the fact that some of the GRPs for which transcripts were not detected in a given species may indeed be present in salivary glands as preformed proteins stored in granules. Nevertheless, this still represents a biological difference involving GRPs that is reflected in the transcription profile. On the other hand, previous work [12] clearly shows that tick salivary glands are not completely "pre-loaded" and ready to secrete when a tick attaches to a host. Indeed, the expression of at least 27 genes encoding secreted proteins increases in salivary glands of female *Ixodes scapularis *ticks after attachment to their host and, interestingly, almost a third (eight) of these encode GRPs. Furthermore, transcripts for GRPs were not observed in salivary glands from unfed females. Kaufman [13] showed that fluid secretion by salivary glands was similar in the females of several species of Ixodidae ticks, including Brevirostrata and Longirostratata ticks suggesting that salivation is similar throughout the Ixodid family [13], i.e., if the presence of 'pre-loaded' granules has a determinant role in salivation, that work would have found differences for distinct tick species, mainly at the early phases of salivation.

Besides analyses performed with the NCBI database, we used the gene ontology (GO) database to categorize the GRP contigs from individual libraries. Results must be interpreted with caution since the GRP sequences are of low complexity and GO categories are still not entirely comprehensive for all biological functions. Nevertheless, differences were seen among the three species of ticks. The GRP transcripts were categorized into GO terms for nine biological processes (Additional file 1); the term "epidermis development" was most frequently assigned to transcripts from heteroxenous ticks (SGFRs with 70.2% of the terms and SGFAc with 26.1% versus 12.1% of terms for SGFRs). Glycine-rich proteins related to epidermal development have also been found in others arthropods, such as the silkworm *Bombix mori *[14]. Interestingly terms related to development of epidermis were the most abundant category of all, assigned to SGFRs (70.2%), a library made from a heteroxenous, Brevirostrata tick. Over half (52.2%) of the terms assigned for SGFAc fell into the category "unknown", reflecting the fact that little information is available about biological characteristics of saliva from *A. cajennense *ticks.

## Phylogenetic analyses of Glycine-rich proteins

### Silk-like proteins

**Figure 2:**
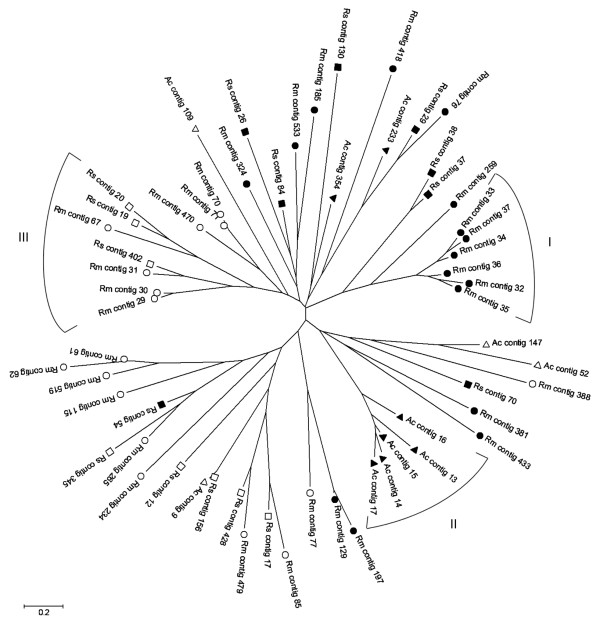
**Dendogram of Glycine-rich proteins of *A. cajennense, R. sanguineus *and *R. microplus *ticks**. The protein sequences were aligned by the Clustal X program (Thompson et al., 1997), and the dendrogram was calculated using the Mega package (Kumar et al., 2004) after 10,000 bootstraps with the neighbor joining (NJ) algorithm. The bar at the bottom represents 20% amino acid substitution. The Roman numerals in the figure indicate the three most distinct clades. Filled symbols are transcripts that presented matches with spider silk proteins and empty symbols are transcripts that displayed matches with cement-like proteins of ticks in Arachnida database (from NCBI). Circle: *R. microplus*; square: *R. sanguineus*; triangle: *A. cajennense*. All similarities (best match against Arachnida database) of transcripts in dendrogram can be observed in Table 2.

Phylogenetic analysis of GRP contigs from the three ticks studied herein shows 3 distinct clades (numbered 1-3) as displayed in Figure 2. Two of them contain a group of contigs that presented similarities with silk proteins of spiders. One was similar to a flagelliform silk protein, (FSP; clade 1), which is a viscous, glue-like silk from orb-weaving spiders of the *Nephila *genus; another was similar to major ampullate spidroin (Masp; clade 2) from *Kukulcania hibern*a*lis*, a cribellate (i.e., it does not produce glue-like adhesive webbing), non-orb-weaving spider. Interestingly, these clades are the most defined in the dendogram, as may be observed through the proximity among members of these clades. Another feature of clades 1 and 2 is that each is formed by contigs from a single species of tick: clade 2 presented contigs only from *A. cajennense *and clade 1 contained contigs only from *R. microplus*, probably for this reason the clades showed better proximity among the members. This finding is compatible with our hypothesis that metastriate ticks presenting with different biological characteristics rely on different types of GRPs. Noteworthy is the fact FSP is an elastic and glue -like adhesive silk [15], a characteristic which could be important for a Brevirostrata monoxenous tick. The third distinct clade has showed similarity with an unknown protein (UK) from *R. haemaphysaloides *and contains contigs from only Brevirostrata ticks, i.e., *R. microplus *and *R. sanguineus*, again a finding that is compatible with our hypothesis. Futhermore, although distinct clades were not formed by the remaining GRP contigs, we noted that contigs segregated into two different patterns: those which presented matches with spider silk proteins (filled symbols) are concentrated in clades that are distant from those that have similarities with cement-like proteins of ticks (open symbols), with the exception of contigs Rs54, Rm388 and Ac52. This finding suggests that these two "major types" of GRPs (silks versus cements) may have different roles during attachment ticks on the host. The similarities found with spider GRPs were also with Masps and FSPs, albeit from different genera of spiders. Interestingly, *A. cajennense *was represented only twice among the cement-like (open symbol) contigs, while two other cement-like contigs from this species grouped with spider silk-like contigs.

**Figure 3:**
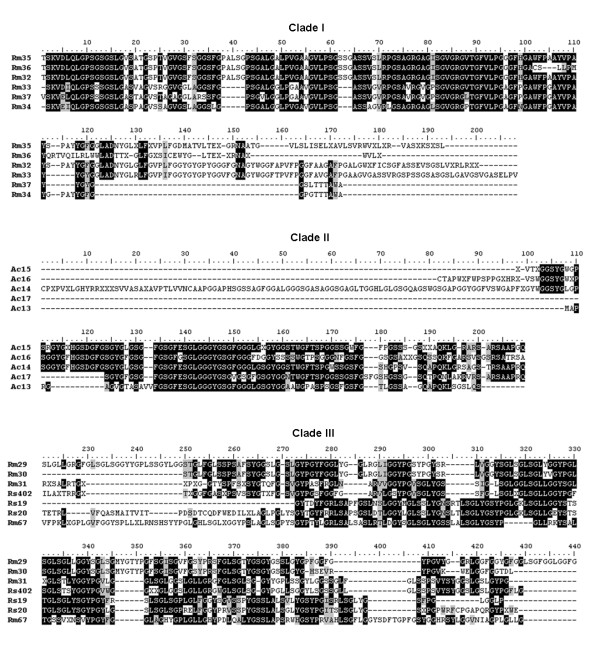
**Multiple alignment of Glycine-rich proteins of *A. cajennense, R. sanguineus *and *R. microplus *ticks**. The transcript protein sequences of clades numbered in figure 3 were aligned in the Clustal X program and analyzed with Bioedit software. Clade 1: transcripts from *R. microplus *that were homologous with flagelliform silk protein of orb-web weaving spiders. Clade 2: transcripts from *A. cajennense *were homologous with major ampullate spidroin protein of non-orb-web weaving spiders. Clade 3: transcripts from *R. sanguineus *and *R. microplus *that were homologous with an "unknown", cement-like protein from *R.. haemaphysaloides*.

A multiple alignment analyses of the three distinct clades (clades 1-3 from Figure 2) showed the level of conservation among contigs, as can be observed in Figure 3. Clade 1, formed by contigs from SGFRm showed the greatest degree of conservation. Sequence alignment of Rm34 and Rm37 show an identity of 78.9% and 81.1% similarity. Rm33 and Rm36 presented the lowest conservation, 33% identity and 35.5% similarity. Contigs of clade 2 formed by SGFAc sequences also displayed low divergence, even the least similar transcript Ac14; Ac13 presented a reasonable equivalence between sequences (25.1% identity and 27% similarity) whereas Ac15 and Ac16 had better conservation, presenting an identity of 56,5% and similarity of 61.4%. Clade 3 did not did not present the same level of conservation as clades 1 and 2, except for contigs Rs 402 and Rm31, which presented a high identity and similarity of 63.4% and 65.9%, respectively.

It was interesting to note the large conserved region visualized in each of the sequence alignments of clades 1 and 2. Five conserved regions are encountered in SGFRm contigs of clade 1 that are identical in the 6 contigs: 1) QLGPS (position 7-11), 2) SGSLG (position 13-17), 3) GVLPSG (position 56-61), 4) SGVGRG (position 82-87) and 5) TGFVLPG (position 89-95); some of these conserved regions could be extend if the charge and chemical proprieties of the residues from different contigs are taken into consideration. In the alignment for clade 1, aspartic acid (D) could change to glutamic acid (E) at residue 5, leucine (L) to isoleucin (I) at residue 6 (both hydrophobic), glycine (G) to serine (S) at residue 12 (both uncharged) and valine (V) to alanine (A) at residue 19 (both hydrophobic) and these amino acids present similar characteristics among each other. The same aspects can be observed for residues 43-61 and 89-98. In addition, when the contigs of clade 1 are compared with the composition of flageliform silk proteins, positions of important residues of silk proteins such as glycine, serine and proline are conserved among them (Additional file 2). Regarding clade 2, two conserved regions are found in all SGFAc contigs, one composed of 5 residues, FGSGF (position 134-138), and a second one with 10 residues, SGLGGGYGSG (position 140-149). It is noteworthy that both regions have glycine (the majority) and serine residues. Again, conserved regions contain glycine and serine residues, two abundant amino acids in spider silk. Alignment of Ac contigs and Masp proteins showed similarity in most positions containing glycine and serine residues, but not in positions with proline residues (Additional file 3). Sheets are formed in secondary structures of silk proteins with repeats containing glycine, serine and alanine, which confer their elastic and strength proprieties [16]. The presence of a proline residue between serine and glycine, as happens in sequences of clade 1, could be important to "interrupt" secondary structures determined by glycine, serine and alanine, promoting acquisition of more elastic and less stiff properties. The mechanical property of elasticity is greater in flagelliform silk proteins of orb-weaving spiders (e.g., the *Nephila *genus) that are made to capture flying prey than in major ampullate spidroin silk proteins (Masp) used in capture threads in less mobile spiders [15,17]. The multiple alignments of clade 3 sequences, which are homologous with an "unknown" protein from *Rhipicephalus haemaphysaloides *did not present conserved regions, perhaps owing to divergence among contigs and many gaps that could not allow long conserved regions. However, it can be observed though shading of the alignments that they present similarities as described before, with regions abundant in glycine and serine.

In addition to the contigs derived from *R. microplus*, *R. sanquineus *and *A. cajennense *analyzed herein and in order to increase the stringency of the test for our hypothesis, we performed a multiple alignment using contigs from the work of Francischetti et al. (2009; http://exon.niaid.nih.gov/transcriptome/tick_review/Sup-Table-1.xls.gz) [18] that reviewed all of the available salivary components of ticks. This work described a superfamily of glycine-rich proteins for argasid and ixodid ticks (mainly Brevirostrata ticks). We observed in this work that Argasid ticks produce only three types out of over four hundred types of GRPs. This maybe due to the fact that Argasid ticks are rapid feeders and complete a blood meal in minutes. We also observed that the majority of the GRPs found in Prostriate ticks (genus *Ixodes*), are collagen-like proteins. This group appears to have a primitive form of attachment among the ixodid ticks [3], presenting an intermediate complexity in this process. Finally, this work showed that in metastriate ticks (from the genera *Amblyomma*, *Dermacentor*, *Rhipicephalus *and *Haemaphysalis*) the GRPs belong to GGY, GYG and metastriate spider-like cement protein families. We therefore excluded analyses of GRPs from Ixodes sp. and Argasidae ticks and selected GRPs from the NR database on NCBI that present similarities with silk-like and cement-like proteins from *A. variegatum*, *A. americanum*, *D. andersoni*, *R. microplus *and *R. appendiculatus *(Sup-Table 1 of Francischetti et al., 2009).

**Figure 4:**
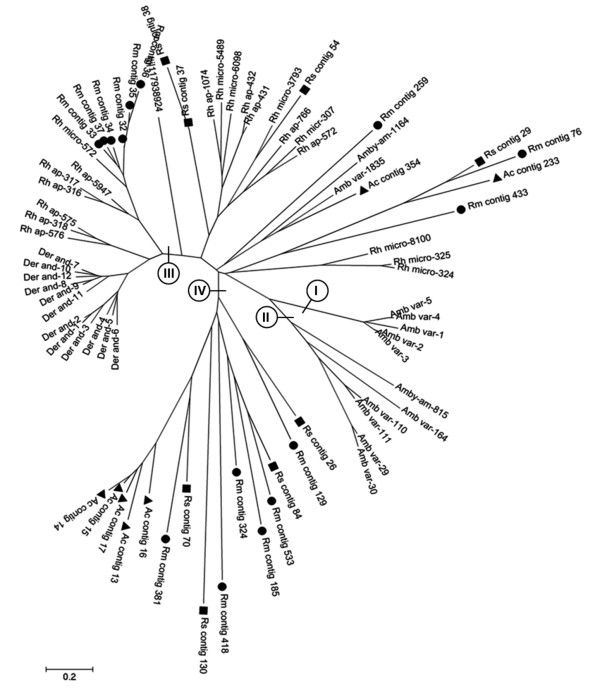
**Dendogram of glycine-rich proteins that present similarities with spider silk proteins**. Transcripts of proteins described in this work (●: *R. microplus*; ■: *R. sanquineus*; ▲: *A. cajennense*) and transcripts of proteins obtained in the catalogue of annotated salivary proteins available in Francischetti et al. (2009) were used to construct phylogenies (Amb var: *A. variegatum*; Amby am: *A. americanum*; Der and: *D. andersoni*; Rh ap: *R. appendiculatus*; Rh micro: *R. microplus*; the numbers refer to contig in Sup-Table1 [18]).

We aligned all sequences similar to silk-like proteins from our libraries and the NR database from Sup-Table 1 (describe in Francischetti et al., 2009) and using the neighbor joining analysis produced the phylogram shown in Figure 4. Sequences generated by the present work are symbolized with a circle (●: *R. microplus*), a square (■: *R. sanquineus *) and a triangule (▲: *A. cajennense*). In addition to our sequences, 45 other transcript sequences of other species ticks are represented: *Dermacentor andersoni *and *R. appendiculatus*, both Brevirostrata, heteroxenous ticks, and *A. variegatum *and *A. americanum*, Longirostrata, heteroxenous ticks. Sequences of *R. microplus *from other sources were also included in the analysis. To the best of our knowledge there are no representatives of Longirostrata, monoxenous ticks that could be included in this analysis. This approach showed that contigs from *Amblyomma *ticks formed distinct clades (1 and 2). These clades did not present similarities with a specific type of flagelliform silk, a similarity consistently found in the Brevirostrata ticks *D andersoni*, *R. microplus*, *R. appendiculatus *and *R. sanguineus*. This can be observed in the most distinct clade formed by these latter species of ticks (clade 3). All sequences from this clade present similarity with flagelliform silk. Moreover, sequences in the dendogram displayed a wide-spread distribution, including a clade formed only by sequences from our libraries (clade 4), showing that we have contributed with diversified sequences encoding glycine-rich proteins from our ESTs database.

### Cement-like proteins

**Figure 5:**
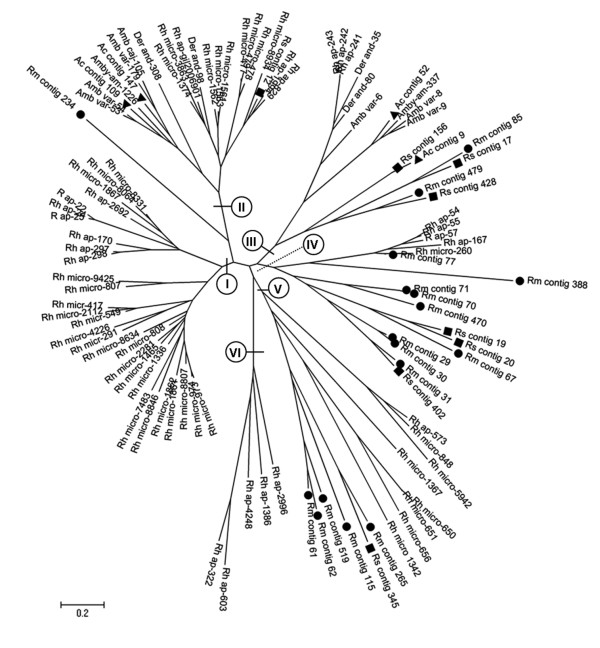
**Dendogram of glycine-rich proteins that present similarities with cement-like proteins**. Transcripts of proteins described in this work (●: *R. microplus*; ■: *R. sanquineus*; ▲: *A. cajennense*) and transcripts of proteins obtained in the annotated catalog of Francischetti et al. (2009) were used to construct phylogenies (Amb var: *A. variegatum*; Amby am: *A. americanum*; Amb caj: *A. cajennense*; Der and: *D. andersoni*; Rh ap: *R. appendiculatus*; Rh micro: *R. microplus*, the numbers refer to contig in Sup-Table1 [18]).

Contigs homologous to so called cement-like proteins were found among each of the three libraries, yet were most abundant in the library derived from *R. microplus *(9 contigs versus two in SGFAc and 1 from SGFRs). The cement-like sequences from the same species analyzed before were aligned and used to generate the phylogenetic tree shown in Figure 5, which showed six distinct clades. Clades 1 and 2 contain cement-like proteins from other Brevirostrata and Longirostrata ticks, indicating the diversity among cement-like proteins. Most sequences from clade 2 presented similarity with the salivary gland-associated protein 64P from *R. appendiculatus*, except sequence Rm 234, which showed similarity with NPL-2 (neuroprotein-like) from *Ixodes pacificus*. Clade 1 contains a subclade with sequences derived exclusively from *R. microplus*. Clade 3, in turn, presented sequences similar to putative cement protein RIM36 from *R. appendiculatus *(Ac 52) and Unknown protein from *R. haemaphysaloides *(Rs 156, 17, 4238; Rm 85, 479 and Ac 9). Clades 4 and 5, aside from illustrating the extensive diversity in the expression of cement-like proteins between the ticks, also indicate the expansion of the genus *Rhipicephalus *showing similarity with cement-like antigen protein from *Haemaphysalis longicornis *and an unknown protein from *R. haemaphysaloides*. Finally, Clade 6 contains sequences derived exclusively from *R. appendiculatus*.

Examination of the best hits to the sequences in the general NCBI database showed that several GRP contigs were significantly homologus to GRPs of plants (Rm 265, Rm 36, Rm 77, Rs 70, Ac 147, Ac 14) vertebrate skin (Rs 26, Rs12, Rs 70, Ac 354, Ac 13, Ac16) nucleic-acid-binding proteins (Rm 32, Ac 233 ) and to the *Mycobacterium tuberculosis *PE-PGRS multigene family (contigs Rm 479, Rm 533, Rm 29, Rs 29). These similarities may also shed light on the biological functions of the tick GRPs. In plants many GRPs form the walls of initially polysaccharide-rich primary water pipes of elongating plant organs [19]. These functions remit to those of the cement cone in Brevirostrata ticks, which forms a continuation of the hypostome that penetrates the host skin. Interestingly, seed plant GRPs can be allergens for vertebrates [20] and similarly tick saliva can elicit local hypersensitivity reactions in immune hosts [21]. GRPs also play a role in regulating permeability and penetration of toxins in insect cuticles [9]. In ticks the cement cone may assist the cuticle of the hypostome in trapping host cells and molecules that are cytotoxic for the parasite. Many secreted salivary GRPs are similar to RNA-binding proteins, which in the tick may participate in modifying the extracellular traps comprised of nucleic acids that can be produced by mast cells and neutrophils [22], which are present in the local inflammatory infiltrate elicited in the host's skin by tick bites. This finding can also explain the significant quantity of transcripts from SGFRm (20.7%, Additional file 1) categorized such as "nucleic acid binding" based on the GO database. Tick GRPs similar to keratins and loricrins, which are major envelope components of terminally differentiated epithelial cells of vertebrate skin [23], may serve as decoys for the host. Interestingly, the Brevirostrata ticks herein analyzed (*R. microplus *and *R. sanguineus*) displayed a greater number of transcripts related to development of epidermis and organization and biogenesis of extracellular matrix based on homologies to the GO database than the Longirostrata tick (*A. cajennense*). Finally, the products of the PE-PGRS multigene family of *M. tuberculosis *form a source of antigenic variation among different strains of this bacterium [24]; in addition PE-PGRS contain many Gly-Ala repeats, which are also present in tick GRPs and which have been shown to inhibit ubiquitin/proteasome-dependent protein degradation in mycobacteria and Epstein-Barr virus [25,26]. Since libraries were constructed from a pool of salivary glands from several individual females, the diversity in contigs of salivary GRPs may reflect the existence of a similar mechanism in ticks.

GRPs present biochemical characteristics that could possibly be involved in stabilizing the tick to its feeding site for long periods due to their putative structural and mechanical functions inferred from the abundance of the amino acid glycine. GRPs may also block host immune system molecules that enter in contact with the tick mouthparts [4]. Many contigs were similar to silk proteins from spiders, such as fibroin, dragline, flagelliform, major ampullate spidroin and flag silks. Each one of these fibers is composed of one or more proteins encoded by the silk fibroin gene family. Spiders draw fibers from dissolved fibroin proteins that are stored in specialized sets of abdominal glands [27]. It is interesting to note that ticks generate silk-like proteins from their salivary glands, while spiders use abdominal glands for this purpose and reserve their salivary glands for production of venom. Tick silk-like GRPs may possibly support mechanical needs (e.g., fixation to host skin), as well as the capture of prey and predators (respectively, blood and cytotoxic leukocytes). Spider silks are being employed as scaffolds for engineering tissues [28] and tick silk-like proteins may be more adequate for this purpose because of the intimate relation of this parasite with its host's skin.

There are other precedents in nature for our finding that the distribution of distinct GRPs correlates with the biology of metastriate ticks. Spiders, which are also Arachnidae, offer a well known example: the architecture and mechanical properties of different spider webs are correlated with the biological characteristics of their spinners, for example, aerial versus terrestrial capture habits. These properties ultimately rely on the specialized functions of different types of silks. Of interest to studies on the evolution of ticks, orb weaving by spiders is monophyletic, having evolved only once and speciation of spiders relates to use of different silks [15]. Genes encoding flagelliform silks were thought to be expressed exclusively by modern orb weaver spiders that make more elastic, gluey webs. However it was recently shown that cribellate orb weavers, which make drier webs, also express flagelliform silk genes [29], albeit in lower quantities. Blackledge and colleagues [15] suggested that an increase in the expression of flagelliform silk genes may have resulted in development of modern orb weavers [15]. Another example refers to the silks produced by salivary glands of simuliid filter-feeding flies. *Simulium noelleri *and *S. ornatum *use silk pads to attach to substrates, the composition of which varies according the requirements of their habitats: *S. noelleri *feeds in lake outlets where weaker currents are found and *S. ornatum *feeds in open waters with stronger currents. Accordingly, there are differences between ageing processes and biochemical composition of the silk pads from these two species, *S. ornatum *presenting the most durable structure [30]. A third and final example is offered by larvae of two species of caddisflies. *Hydropsyche angustipennis *spins hiding tubes and catching nets that collect food in water currents; larvae of *Limnephilus decipiens *use silk fiber only for stitching fragments of grass into hiding and pupation cases. The composition of the silk fibers from these species differed by the arrangement of motifs in higher order repeats and by the presence of species-specific motifs. Although the amounts of glycine are similar, the H-fibroin of *H. angustipennis *presents proline containing motifs, whereas *L. decipiens *presents a highly charged motif, EEGRRR [31].

## Conclusions

In the present work the differences observed for distribution of glycine-rich proteins were related to the number of hosts visited (i.e., if the species is monoxenous or heteroxenous) and to the anatomy of mouthparts (long or short hypostome) of three species of metastriate ticks. All ixodid ticks, with the exception of some Prostriate, present a strategy for attachment, but it differs among them. The species from the genus *Amblyomma*, which belongs to the Longirostrata ticks, secrete a casing around their long, fully inserted hypostome. In ticks from the Brevirostrata group, which includes species from the genus *Rhipicephalus*, the mouthparts are short and barely penetrate in epidermis [3], so a larger cement cone, from which GRPs have been purified [4], is necessary and is deposited in the upper layers of their host's skin. Thus, it seems that the two Brevirostrata ticks, *R. microplus *and *R. sanguineus*, need to express more glycine-rich proteins than the Longirostrata tick, *A. cajennense*, in order to compensate for the small size of mouthparts and for the superficial fixation at the site of attachment. Furthermore, *R. microplus *is monoxenous and *R. sanguineus *is heteroxenous and comparisons made between these ticks show that the former presents the greatest diversity of glycine-rich proteins, possibly because it is a one-host tick that feeds uninterrupted for many days until completion of its life cycle and, therefore, has greater demands for sustaining its attachment on host skin.

Contigs of salivary glands for several other species of ticks have also been examined. While the relative abundance of transcripts coding for glycine-rich proteins cannot be accurately compared between salivary gland libraries constructed in different laboratories and undergoing different biological situations (for example, infection and feeding time, number of salivary glands used or if whole body ticks were used, etc), it is still noteworthy that annotation of the transcriptomes of salivary glands from female *I. scapularis *and *I. pacificus *indicate that prostriate ticks do not rely on glycine-rich proteins as heavily as metastriate ticks for their attachment to hosts or for other biological functions [32,12]. On the other hand, salivary glands of females of *D. andersoni*, a metastriate, heteroxenous, Brevirostrata tick, also contain abundant transcripts for GRPs: of the 30 contigs containing the most abundantly expressed ESTs in salivary glands of females of *D. andersoni*, 9 presented similarities to glycine-rich proteins and contained from 21 to 5 ESTs each [33].

In conclusion, our findings furnish preliminary evidence to support the hypothesis that species of ticks with differences in the anatomy of their mouthparts and in the number of hosts they infest during their biological cycle rely on different types and quantities of glycine-rich proteins. This hypothesis must be further tested by expanding these observations to a larger number of species, by experimental approaches such as RNA interference of expression of selected GRPs and by characterization of isolated GRPs. The data suggests that prostriate ticks rely on their elongated barbed hypostome mouthparts and make shallow cement cones, while the metastriate ticks rely on a larger and deeper cement cone possibly to compensate their relatively smaller mouth parts [3]. The number of hosts visited by ticks during the parasitic stage of their life cycle also requires adaptations. According to Balashov (1972) [34] and Hoogstraal and Kim (1985) [35] there was a transition from the three host to the two and one host cycle in *Hyalomma *and in Rhipicephalinae species of ticks. The biological characteristic of having a single host is regarded as an adaptation of this immobile ectoparasite to large nomadic animals since ixodid ticks die when they are unable to find a host. Monoxenous ticks are thus better adapted to open environments inhabited by large, grazing ungulates. The ability to molt on the vertebrate reduces the number of necessary encounters and thus increases chances for tick survival.

In addition to elucidating the biology of tick salivary proteins, the information contained in this work is relevant for the development of vaccines that target GRPs of ticks and that aim for protection against a broad range of species. The approach undertaken in this work can subsidize the choice of the different GRPs present in tick salivary glands for evaluation as protective antigens.

## Methods

### Ticks

Adult female ticks of *Rhipicephalus *(Boophilus) *microplus*, *Rhipicephalus sanguineus *and *Amblyomma cajennense *were collected from naturally infested vertebrate hosts; cattle, dogs and horses, respectively. Samples were collected as to cover the feeding process until the phase of rapid engorgement. Ticks of different sizes, but always ≤ 4 mm in body length (before the rapid engorgement phase of feeding; approximately three to four days post attachment) were used for salivary gland dissection to avoid degeneration of salivary gland proteins [36,37]. Ticks were collected from a sample of several hosts and over a period of two to five days and, once removed from the hosts, salivary glands were immediately dissected; a total of 20-30 ticks were used per library. Glands were briefly washed in ice-cold 1X PBS and immediately stored in RNA later storage solution at 4°C for 24 hours and (Ambion, Austin, TX, USA) then transferred to -80°C for long term storage.

### Extraction of mRNA and cDNA library synthesis

Poly A+ mRNA from tick salivary glands was isolated using the Micro-Fast Track™ 2.0 mRNA isolation kit (Invitrogen, Carlsbad, California) following the manufacturer's instructions. mRNA (similar concentrations for all samples) was used to construct the cDNA library using the vector TriplEx2 according to the instructions for the SMART™ cDNA Library Construction kit (Clontech, Palo Alto, California) with some modifications [38] and packaged into lambda phage using the Gigapack^® ^III Gold Packaging Extract (Stratagene, La Jolla, California).

The phage sample was used as a template for a PCR reaction to randomly amplify cDNAs. The primers used for this reaction were sequences from the TriplEX2 vector. PT2F1 (5' -AAG TAC TCT AGC AAT TGT GAG C-3') is positioned upstream of the cDNA of interest (5' end), and PT2R1 (5'-CTC TTC GCT ATT ACG CCA GCT G-3') is positioned downstream of the cDNA of interest (3' end). The cleaned PCR product was used as a template for a cycle-sequencing reaction using the Big Dye kit (Applied BioSystems, Foster City, California).The primer used for sequencing, PT2F3 (5'-TCT CGG GAA GCG CGC CAT TGT-3') is upstream of the inserted cDNA and downstream of the primer PT2F1. Sequencing reactions were performed in one direction only on a Gene Amp PCR System 9700 (Applied Biosystems, Foster City, California).

### Bioinformatic tools

Detailed description of the bioinformatic treatment of the data can be found elsewhere with some modifications [38,12]. The programs used were written in Visual Basic 6.0 (Microsoft, Redmond, Washington) by one of us (JMR). Briefly the ESTs (raw sequences) were trimmed of primer and vector sequences, clustered into related groups, based on homology, using the BLASTN algorithm (minimum identity of 81 nucleotides over 90 nucleotides) [39], and then assembled and aligned using the CAP3 assembler [40]. The consensus sequences and singletons resulting from the CAP3 assembler were compared to the Non-Redundant (NR) protein database of the NCBI; a customized protein database containing all Arachnida sequences available on Genbank, and the Gene Ontology (GO) database [41] using the BLASTX algorithm (downloaded from an executable file obtained from the NCBI FTP site [39]. Since libraries were constructed in a unidirectional orientation, BLASTX results were only considered if they were on the positive sense strand. A cut-off E-value of 10^-3 ^was considered for annotation. All sequences were translated into three Sequences containing >5% non-assigned nucleotides (Ns) or final length of less than 100 nt were removed from the analysis and assumed to be of poor quality. The final output was piped into a tab-delimited file imported into an Excel (Microsoft Excel Analysis Tools, Seattle, WA) spreadsheet. We used the χ^2 ^test and Fisher test to analyze differences in the distribution of ESTs in the different libraries. Phylogenetic analysis of glycine-rich contigs was conducted by first aligning sequences obtained from our cDNA library analysis with published GRP sequences recently cataloged by Francischetti et al. (2009) [18] as well as silk protein sequences obtained from Genbank, using ClustalX Sequence Alignment program [42]. Alignments were manually refined using BioEdit sequence editing software [43]. Phylogeneic associations were determined using neighbour joining (NJ) analysis (Mega 4.0 [44]). Node support of each clade was evaluated using bootstrap analysis (1000 replicates).

## Accession numbers

All sequences are deposited in dbEST (Expresses Sequence Tags database) of GenBank (NCBI). SGFAc: gi 224366827 - gi 224366849; SGFRm: gi 224366850 - gi 224366907 and SGFRs: gi 224366908 - gi 224366954.

## Abbreviations

SGF: stands for salivary glands of female ticks; Rm: for *Rhipicephalus *(Boophilus) *microplus*; Rs: for *Rhipicephalus sanguineus*; Ac: *Amblyomma cajennense*. Thus, the abbreviations SGFRs, SGFRm and SGFAc mean, respectively, cDNA library of salivary glands from feeding female ticks of *Rhipicephalus sanguineus*, *Rhipicephalus *(Boophilus) *microplus *and *Amblyomma cajennense*.

## Authors' contributions

SRM and EA constructed the libraries, performed the bioinformatic treatment of the sequences, performed the analyses, including phylogenetic and statistical analyses, and drafted the manuscript; JMA assisted with the bioinformatic treatment of the sequences, the analyses and preparation of the manuscript; JMR assisted with the bioinformatic treatment of the sequences and analyses; JGV assisted with the strategy of library construction, LGB and GRG constructed the libraries; SP, RB and MPJS assisted in the analyses; BRF and IKFMS participated in the study's coordination and drafted the manuscript; IKFMS conceived the study. All authors read and approved the final manuscript.

## Supplementary Material

Additional file 1 **Biological categories for GRP contigs obtained from Gene Ontology**. Assignment of Gene Ontology (GO) biological process terms to the glycine-rich proteins from libraries of female salivary glands of *A. cajennense*, *R. sanguineus and R. microplus *ticks. Click here for file

Additional file 2 **Comparison between sequences of *R. microplus *ticks and *Nephila *spiders**. Multiple alignment of glycine-rich proteins *R. microplus *ticks from Clade 1 (Figure 2) and flagelliform silk proteins from *Nephila *genus obtained from GenBank [acession numbers: Nc-FSP871 (AAC38846.1), Nm-FSP1884 (AAF36091.1) and Nc-FSP2249 (AAF36090.1)]. Click here for file

Additional file 3 **Comparison between sequences of *A. cajennense *ticks and *Kukulcania *spiders**. Multiple alignment of glycine-rich proteins of *A. cajennense *ticks from Clade 2 (Figure 2) and major ampullate spidroin from *Kukulcania *genus obtained from GenBank [accession numbers Kh-Masp2.1 (AAT08434.1) and Kh-Masp2.2 ( AAT08435.1)]. Click here for file
