# Supplementary figures and images for: The expression of genes coding for distinct types of glycine-rich proteins varies according to the biology of three metastriate ticks, Rhipicephalus (Boophilus) microplus, Rhipicephalus sanguineus and Amblyomma cajennense
Source: BMC Genomics. 2010 Jun 8;11:363. doi: 10.1186/1471-2164-11-363 (PMC2901319; doi:10.1186/1471-2164-11-363)

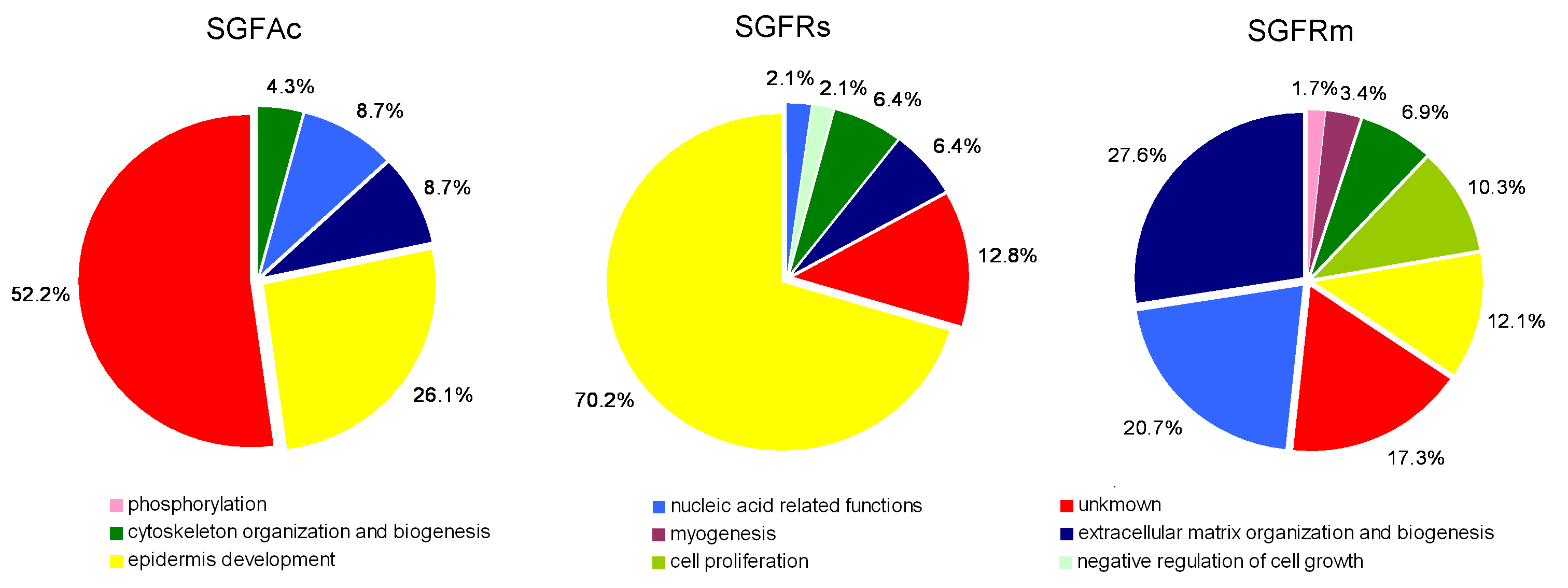

Supplement: Additional file 1 — Biological categories for GRP contigs obtained from Gene Ontology. Assignment of Gene Ontology (GO) biological process terms to the glycine-rich proteins from libraries of female salivary glands of A. cajennense, R. sanguineus and R. microplus ticks. [file 1471-2164-11-363-S1.TIFF]

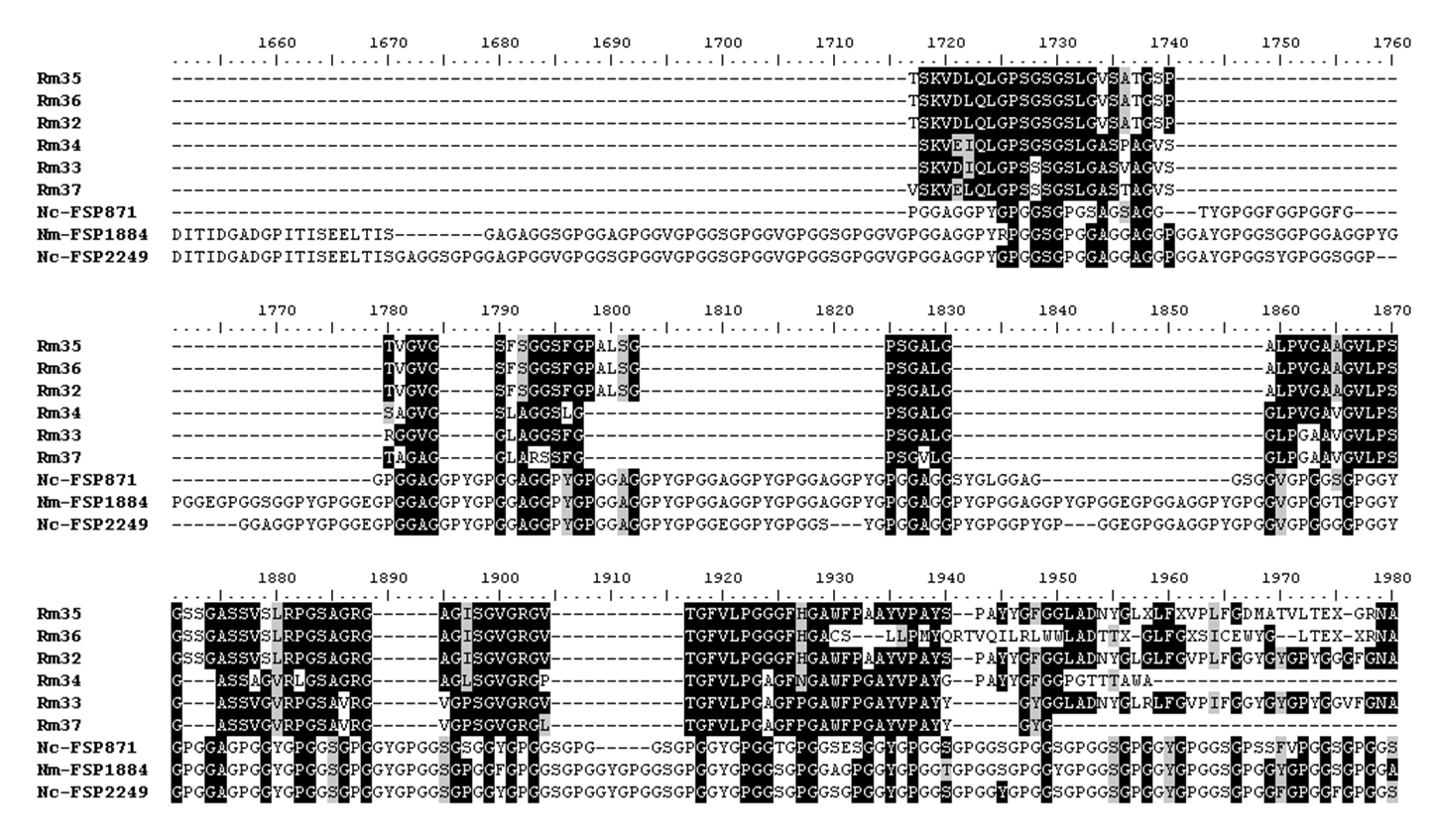

Supplement: Additional file 2 — Comparison between sequences of R. microplus ticks and Nephila spiders. Multiple alignment of glycine-rich proteins R. microplus ticks from Clade 1 (Figure 2) and flagelliform silk proteins from Nephila genus obtained from GenBank [acession numbers: Nc-FSP871 (AAC38846.1), Nm-FSP1884 (AAF36091.1) and Nc-FSP2249 (AAF36090.1)]. [file 1471-2164-11-363-S2.TIFF]

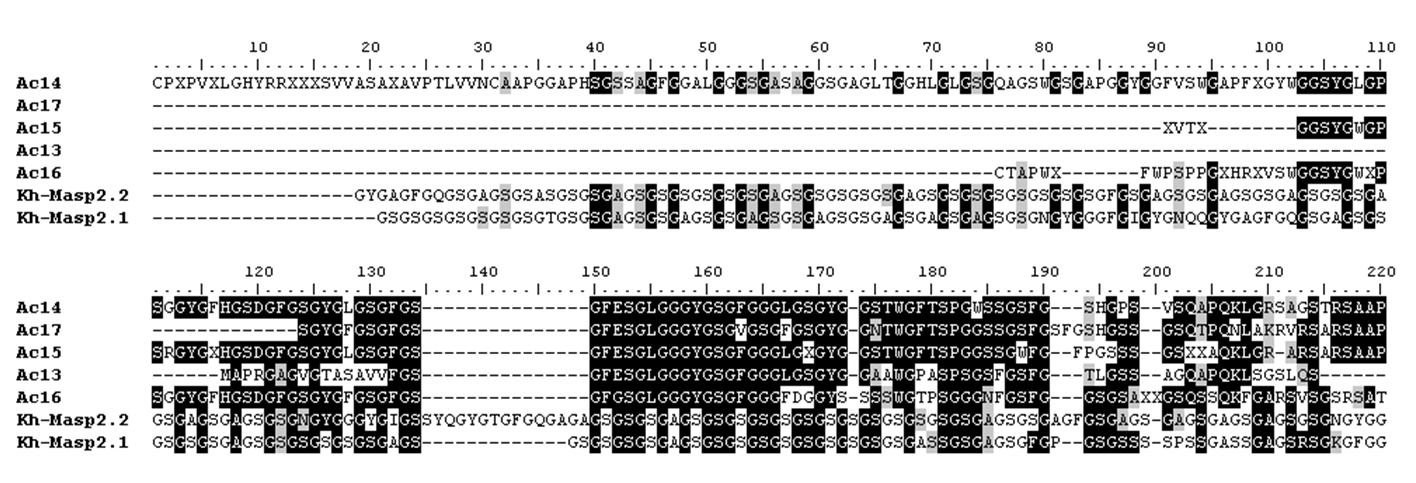

Supplement: Additional file 3 — Comparison between sequences of A. cajennense ticks and Kukulcania spiders. Multiple alignment of glycine-rich proteins of A. cajennense ticks from Clade 2 (Figure 2) and major ampullate spidroin from Kukulcania genus obtained from GenBank [accession numbers Kh-Masp2.1 (AAT08434.1) and Kh-Masp2.2 ( AAT08435.1)]. [file 1471-2164-11-363-S3.TIFF]
